# Genetic Variations and Haplotype Diversity of the *UGT1* Gene Cluster in the Chinese Population

**DOI:** 10.1371/journal.pone.0033988

**Published:** 2012-04-13

**Authors:** Jing Yang, Lei Cai, Haiyan Huang, Bingya Liu, Qiang Wu

**Affiliations:** 1 Key Laboratory of Systems Biomedicine (Ministry of Education), Center for Comparative Biomedicine, Institute of Systems Biomedicine, Shanghai Jiao Tong University, Shanghai, China; 2 State Key Laboratory of Oncogenes and Related Genes, Shanghai Cancer Institute, Renji Hospital, School of Medicine, Shanghai Jiao Tong University, Shanghai, China; Midwestern University, United States of America

## Abstract

Vertebrates require tremendous molecular diversity to defend against numerous small hydrophobic chemicals. UDP-glucuronosyltransferases (UGTs) are a large family of detoxification enzymes that glucuronidate xenobiotics and endobiotics, facilitating their excretion from the body. The *UGT1* gene cluster contains a tandem array of variable first exons, each preceded by a specific promoter, and a common set of downstream constant exons, similar to the genomic organization of the protocadherin (*Pcdh*), immunoglobulin, and T-cell receptor gene clusters. To assist pharmacogenomics studies in Chinese, we sequenced nine first exons, promoter and intronic regions, and five common exons of the *UGT1* gene cluster in a population sample of 253 unrelated Chinese individuals. We identified 101 polymorphisms and found 15 novel SNPs. We then computed allele frequencies for each polymorphism and reconstructed their linkage disequilibrium (LD) map. The *UGT1* cluster can be divided into five linkage blocks: Block 9 (*UGT1A9*), Block 9/7/6 (*UGT1A9*, *UGT1A7*, and *UGT1A6*), Block 5 (*UGT1A5*), Block 4/3 (*UGT1A4* and *UGT1A3*), and Block 3′ UTR. Furthermore, we inferred haplotypes and selected their tagSNPs. Finally, comparing our data with those of three other populations of the HapMap project revealed ethnic specificity of the *UGT1* genetic diversity in Chinese. These findings have important implications for future molecular genetic studies of the *UGT1* gene cluster as well as for personalized medical therapies in Chinese.

## Introduction

The adaptive immune system, central nervous system (CNS), and chemical defense system in vertebrates require tremendous molecular diversity to defend against viruses and bacteria, to specify complex neuronal connectivity, and to remove numerous small hydrophobic chemicals from the body, respectively [1], [2], [3]. The vertebrate genomes generate the required molecular diversity in these systems through gene duplication, gene conversion and transposition, somatic mutation and DNA rearrangement, alternative splicing, promoter usage, and polyadenylation, copy number variation, as well as single nucleotide polymorphism of the clustered immunoglobulin, *Pcdh* (*PCDHA*, MIM# 604966; *PCDHB*, MIM# 604967; *PCDHG*, MIM# 604968), and *UGT1* genes [2], [4], [5], [6], [7], [8], [9]. These gene clusters are organized into variable and constant regions [4], [10], [11]. Each cluster contains a large number of highly-similar variable exons organized in a tandem array followed by a single set of downstream constant exons [4], [10], [11].

In the adaptive immune system, somatic mutation and DNA rearrangement of the immunoglobulin and T-cell receptor gene clusters play a critical role in generating vast molecular diversity required for defense against unlimited number of foreign antigens [4]. In the central nervous system, alternative promoter usage and alternative splicing play an essential role in generating tremendous molecular diversity of neural cell adhesion Pcdh proteins [12], [13]. These Pcdhs may specify diverse neuronal connectivity in the brain that is required to control complex human behavioral repertoire such as language, tool use, emotion, empathy, culture learning, and consciousness [2], [10]. In addition, positive selection and gene conversion of clustered *Pcdh* genes also increase the diversity of Pcdh proteins [6], [14]. Finally, species-specific gene duplications and exon mutations suggest that birth-and-death evolution plays an important role in the dynamic evolvement of the clustered *Pcdh* genes [14].

**Figure 1:**
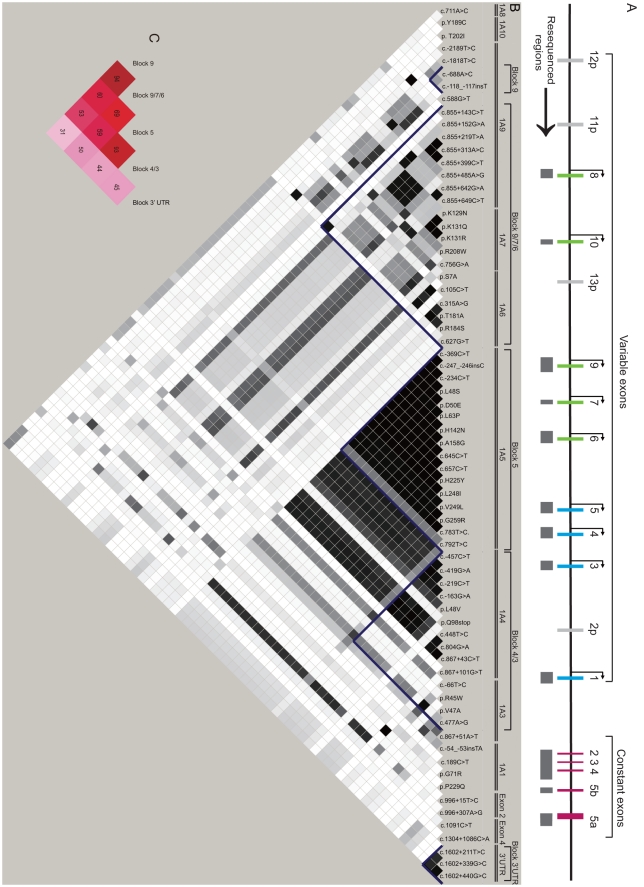
Polymorphisms and linkage analyses of the *UGT1* gene cluster in Chinese Population. (**A**) The genomic structure of the *UGT1* gene cluster. The resequenced regions are indicated by gray boxes under each exon. The diagram is drawn to scale. (**B**) Pairwise Linkage disequilibrium (LD) analysis and haplotype block reconstruction in the entire *UGT1* locus. The polymorphisms are in the same order as the genomic organization on chromosome 2. The extent of the LD is represented by different shades of gray. The blue lines are the boundaries of haplotype blocks. (**C**) Multiallelic LD analysis of the five haplotype blocks in the *UGT1* locus. The numbers (%), generated by the Haploview 4.1, represent the D’ values between any two haplotype blocks.

In the vertebrate chemical defense system, diverse phase II drug-metabolizing enzymes, which are encoded by the *UGT1* gene clusters, glucuronidate a wide range of endobiotic and exobiotic hydrophobic chemicals, converting them into hydrophilic molecules [15], [16]. The vertebrate *UGT1* cluster is organized into multiple variable genes arrayed in tandem and a single set of constant exons (Fig. 1A) [7], [11], [16], [17], [18], [19]. Each of the *UGT1* variable exons is alternatively spliced to the common set of constant exons to produce diverse mRNA and protein isoforms [11], [17], [18]. The encoded UGT1 enzymes contain an N-terminal Rossmann domain that recognizes numerous acceptor substrates and a C-terminal Rossmann domain that binds to the UDP glucuronic acid (UDPGA) donor [7], [20], [21]. The acceptor substrates sit in a pocket in the N-terminal Rossmann domain encoding by variable exons, and the donor substrate UDPGA lies in the C-terminal Rossmann domain encoded by the constant exons [7], [21], [22], [23]. The acceptor binding pocket is surrounded by four hypervariable regions consisting of very diverse residues [7]. UGT1 enzymes catalyze the transfer of the glucuronic acid moiety from UDPGA to hydrophobic acceptor substrates to increase their hydrophilicity. Thus, glucuronidation by UGT1 enzymes is an important pathway for detoxification of environmental toxins, biotransformation of therapeutic drugs, and metabolism of endobiotics.

In addition to the *UGT1* diversity generated by alternative splicing, *UGT1* genetic diversity, such as single nucleotide polymorphisms (SNPs), also affects their enzymatic activities [15]. Some *UGT1A1* SNPs have been associated with hyperbilirubinemic diseases such as Crigler-Najjar syndrome types I and II (CNI, MIM #218800 and CNII, MIM #606785), Gilbert syndrome (GS, MIM #143500), as well as severe side effects of medicines, such as diarrhea and neutropenia of the colorectal cancer drug irinotecan [15], [24], [25], [26]. Thus, the *UGT1* SNPs may be used as biomarkers for assessing individualized disease risk and personalized medical therapy. However, owning to the overlapping substrate specificity and the differential linkage disequilibrium between SNPs, it is challenging to identify functional *UGT1* SNPs. Because haplotypes have greater power and are more appropriate to be used for genotype-phenotype correlations than individual SNPs [27], it is necessary to perform haplotype analyses of the entire *UGT1* locus in a large population sample.

To systematically analyze the haplotype architecture of the *UGT1* locus, we resequenced the *UGT1* gene clusters, including nine functional genes (*UGT1A1*, MIM 191740; *UGT1A3*, MIM 606428; *UGT1A4*, MIM 606429; *UGT1A5*, MIM 606430; *UGT1A6*, MIM606431; *UGT1A7*, MIM 606432; *UGT1A8*, MIM 606433; *UGT1A9*, MIM 606434; and *UGT1A10*, MIM 606435) and their flanking regions, in a large sample of the Chinese population. We identified 101 polymorphisms in this Chinese cohort, including 15 novel ones. In addition, we reconstructed the LD map of the whole *UGT1* locus. Moreover, we inferred haplotypes at the structural levels of *UGT1* variable exons, linkage blocks, and the entire *UGT1* locus. TagSNPs for each of the inferred haplotype were also identified. Finally, we compared the SNP frequency, LD map, and haplotype of the Chinese population with those of the Japanese, Caucasian, and African populations. Our results reveal an ethnic-specific pattern of molecular diversity of the clustered *UGT1* genes. This work provides an important insight into the genetic variation and genomic architecture of the *UGT1* cluster and lays a solid foundation for further pharmacogenomics studies in Chinese.

## Materials and Methods

### DNA Samples

Peripheral blood samples from 253 unrelated healthy Chinese individuals were obtained from the Henan Regional Hospital after their use in routine physical examinations. Total genomic DNA was isolated from the blood samples by using the Promega Wizard Genomic DNA Purification Kit. The use of these samples was approved by the hospital. Written informed consent was obtained from participants. The study was reviewed and approved by the Institutional Ethics Committee of Shanghai Jiao Tong University.

### *UGT1* Sequencing

We screened nine *UGT1* first exons, five common exons, and their adjacent regulatory and intronic regions by sequencing 12 PCR-amplified regions of 253 individuals covering a total length of about 17.7 kb (Fig. 1A and Table S1). The gene-specific primer pairs were designed according to the reference sequence AF297093.1 (Table S1) [28]. The PCR amplification was performed in a 20-µl reaction containing 10 ng of genomic DNA, 2 µl of 10 × PCR buffer, 2 µl of 2.5 mM dNTPs, 0.25 µmol of each primer, and 1 unit of Taq polymerase. After a hot start at 94°C for 3 min, 35 cycles of 94°C, 30 seconds for denaturing, 50–65°C (specific annealing temperatures for PCR reactions are indicated in Table S1), 30 seconds for annealing, and 72°C, 50 to 90 seconds for extension were performed. The final extension was incubated at 72°C for 7 min. Each of the PCR fragments was gel-purified and sequenced in two opposite directions (Fig. 1A and Table S1). Sequences were analyzed with the Vector NTI Advance 10 software (Invitrogen).

The levels of the pairwise linkage disequilibrium (LD) were calculated with HAPLOVIEW 4.1 software [29], [30] for all of the polymorphisms identified except those with the frequency <0.005 or the p-value of Hardy-Weinberg equilibrium (HWE) <0.05. The density of the color reflects the LD value (r^2^) with the denser the color, the higher the LD of the pair of markers (Fig. 1B). The haplotype blocks were reconstructed for all of polymorphisms except those with the minor allele frequency (MAF) <0.005 or a p-value <0.05 as determined by HAPLOVIEW 4.1 [29], [30]. We also confirmed these results by using the GEVALT 2.0 software [31].

### Haplotype Reconstruction and TagSNPs Selection

The *UGT1* haplotypes were inferred with the Bayesian statistical method by using the Phase 2.1.1 program [32]. The TagSNPs were selected with the STAMPA program of the GEVALT 2.0 software [31]. The minimal subsets of SNPs were selected as the tagSNPs when their prediction accuracy is more than or equal to 99% to represent all of the SNPs.

### HapMap Analysis

We downloaded the genotyping data from the HapMap database of three other populations: the Japanese in Tokyo area, Japan (JPT, 45 unrelated individuals), the Caucasian with northern and western European ancestry from Utah, United States (CEU, 30 trios), and the Yoruba people in Ibadan, Nigeria (YRI, 30 trios). We compared our data of the Chinese Han in Henan province (CHH) with those of the three HapMap populations.

## Results

### Analyses of Polymorphisms of the *UGT1* Gene Cluster in a Sample of Chinese Population

To analyze ethnic-specific patterns of human variations of the *UGT1* gene cluster, we screened a set of 12 regions including promoters, exons, introns, and 3′ UTR of the nine functional *UGT1* genes for polymorphisms in a population of 253 unrelated healthy Chinese individuals (Fig. 1A). To identify the complete SNP repertoire in this cohort, we included the *UGT1A5* gene even though the encoded enzyme has very low activity [33]. We found a total of 101 polymorphisms (Table S2). Ninety of them are in the variable region and 11 are in the constant region. We observed only one polymorphism (UGT1A1*63 of exon 4, i.e. UGT1A1.63) within an exon of the constant region. However, we identified 10 SNPs in the noncoding sequences of the constant region. Among the 101 detected polymorphisms, 14 are not in the Hardy-Weinberg equilibrium, and 18 have a frequency <0.5%, which were excluded in the following LD analysis. Interestingly, one SNP affecting the protein sequence is not in HWE (pS141C).

We identified 15 novel polymorphisms (Table S2), all of which, except the p.S141C of *UGT1A10* (p  =  0.004), confirm to the Hardy-Weinberg equilibrium. Ten of these polymorphisms are in the coding sequences, two are in the promoter regions, two are in the intronic regions, and one is in the 3′ UTR. Six of the 10 novel polymorphisms in the coding region are nonsynonymous. They are p.N209D (MAF  =  0.004) of *UGT1A8*, p.S141C (MAF  =  0.004) and p.Y189C (MAF  =  0.006) of *UGT1A10*, p.Q182R (MAF  =  0.002) of *UGT1A9*, and p.P10L (MAF  =  0.002) and p.T78S (MAF  =  0.004) of *UGT1A4*. Among these 15 novel polymorphisms, 11 are rare SNPs, as judged by MAF <0.005. Two of the four with MAF >0.005 are in the coding region and the two in the noncoding region (c.*−2189T>C of *UGT1A9* and c.*1304+1086C>A of intron 4 in the constant region) have relatively high frequencies of MAF > 0.014 (Table S2).

We identified three polymorphisms of nucleotide insertions in the promoter regions, −118insT resulting in −118T10 of *UGT1A9*, −53ins(TA) resulting in −53(TA)7 of *UGT1A1*, and −246insC resulting in −246C5 of *UGT1A5* (Table S2). The former two are known to influence the *UGT1* gene expression [34], [35]. We however did not observe the UGT1A1*36 (−53(TA)5) and UGT1A1*37 (−53(TA)8) alleles at this site, which were previously reported in the Caucasian and African populations [36].

### LD Analyses and Haplotype Block Reconstruction

We performed pairwise LD analyses for all of the 69 *UGT1* polymorphisms in HWE and with the MAF >0.005 (Fig. 1B). We used the algorithm of confidence intervals to reconstruct the haplotype block [29]. A strong LD was defined as having a one-sided upper 95% confidence bound on D’ as >0.98 and a lower bound is above 0.7 [29]. A block is reconstructed if 95% of informative SNP pairs are in “strong LD”.

The *UGT1* locus can be divided into five haplotype blocks: Block 9 with two polymorphisms (c.−688A>C and UGT1A9*1b) in the promoter region of *UGT1A9*; Block 9/7/6, composed of the intronic SNPs of *UGT1A9* and the coding SNPs of *UGT1A7* and *UGT1A6*, spanning a large region of about 20 kb, which is quite similar to those of the Japanese and Caucasian populations [9], [37]; Block 5, consisting of three polymorphisms in the promoter region and 13 polymorphisms in the coding region of the *UGT1A5* gene, spanning only about 1 kb; Block 4/3, consisting of SNPs of *UGT1A4* and *UGT1A3*, spanning about 11 kb, which has not been observed in other populations; and Block 3′ UTR, composed of three SNPs, 1A1*76, 1A1*78, and 1A1*79, in the 3′ UTR region. We did not observe that the *UGT1A8* and *UGT1A10* SNPs belong to one block as reported in the Japanese population [37] and that the SNPs of *UGT1A3* and *UGT1A1* genes are in one block as reported in the Caucasians [9], [38].

To reveal more clearly the boundaries of the LD blocks, we reconstructed the haplotype blocks using only those polymorphisms in HWE and with the MAF >0.05, excluding all of the polymorphisms with the MAF between 0.005 and 0.05. This did not affect the haplotype block structure with the exception of the Block 9/7/6. This block now excludes three polymorphisms: c.855+143C>T (MAF  =  0.026) and c.855+152G>A (MAF  =  0.166) of *UGT1A9*, and c.627G>T (MAF  =  0.024) of *UGT1A6* (data not shown).

In addition to the paired-polymorphism linkages in the same block, we also observed long-distance LDs among different blocks. For example, the polymorphisms of the *UGT1A5* and *UGT1A4* genes, though in separate blocks, have a strong linkage. This strong linkage was represented by the rectangular shape between Block 5 and the SNPs of *UGT1A4* (0.880< D’ <0.950; 0.800<R^2^ <0.890) (Fig. 1B). Moreover, there is a strong LD between Block 9 and Block 9/7/6, represented by a small rectangle (0.680< D’ <0.970; 0.180< R^2^ <0.800) (Fig. 1B). Finally, there is a relatively strong linkage between the intronic SNPs c.855+152G>A, c.855+642G>A, c.855+649C>T of *UGT1A9*, the c.756G>A of *UGT1A7*, and many polymorphisms of *UGT1A5* and *UGT1A4* genes (0.810< D’ <0.880; 0.600< R^2^ <0.690) (Fig. 1B).

We also noted that one intronic SNP downstream of the constant exon 2 c.996+307A>G has a strong linkage with many polymorphisms of *1A5* and *1A4* (0.920< D’ <0.960; 0.780 <R^2^ <0.850). The *1A5* polymorphisms also have a moderate LD with two *1A3* alleles: c.-66T>C and 1A3*1c (0.890< D’ <0.920; 0.430 <R^2^ <0.770).

To investigate the interblock linkage, we performed a pairwise multi-allelic LD analysis by using the HAPLOVIEW (Fig. 1C) [30]. This analysis confirmed the strong linkage between Block 9 and Block 9/7/6 (D’  =  0.94; R^2^  =  0.157), and also between Block 5 and Block 4/3 (D’  =  0.93; R^2^  =  0.118) (Fig. 1C).

The human *UGT1A1* gene plays an important role in the metabolism of the endobiotic bilirubin and exobiotic irinotecan [26], [35]. Thus, its genotyping has been used in predicting jaundice and personalized treatment of colorectal cancers. We identified sets of polymorphisms of the entire *UGT1* locus linked with three important *UGT1A1* polymorphisms. First, the *1A1* promoter insertion polymorphism c. −54_−53insTA (1A1*28), associated with the Gilbert Syndrome, has a moderate to high LD with the *1A3* polymorphisms (C.−66T>C, R^2^  =  0.233; 1A3*10a, R^2^  =  0.740; 1A3*1c, R^2^  =  0.236; c.867+51A>T, R^2^  =  0.314) and one *1A5* allele, c.792T>C (D’  =  0.805, R^2^  =  0.205) (Fig. 1B). However, the 1A1*28 has extensive higher LDs with a vast number of polymorphisms in the *UGT1A3*, *UGT1A6*, *UGT1A7*, and *UGT1A9* first exons in the Caucasian population [9], [38]. Second, the *1A1* c.211G>A (1A1*6) has a low to moderate LD with some SNPs of the *1A9*, *1A7* and *1A6* genes (Fig. 1B). The 1A1*6 allele has a reduced glucuronosyltransferase activity for SN−38 in the Japanese cancer patients [39], [40]. Finally, the UGT1A1*27, also associated with the Gilbert Syndrome, has a complete linkage with the *1A4* intronic SNP c.867+101G>T (1A4*1d) (D’  =  1, R^2^  =  1) (Fig. 1B), suggesting that, in addition to 1A1*27, the 1A4*1d can be used as a genotyping marker for the Gilbert Syndrome.

### Haplotype Reconstruction and TagSNPs Selection for the *UGT1* Locus

**Figure 2:**
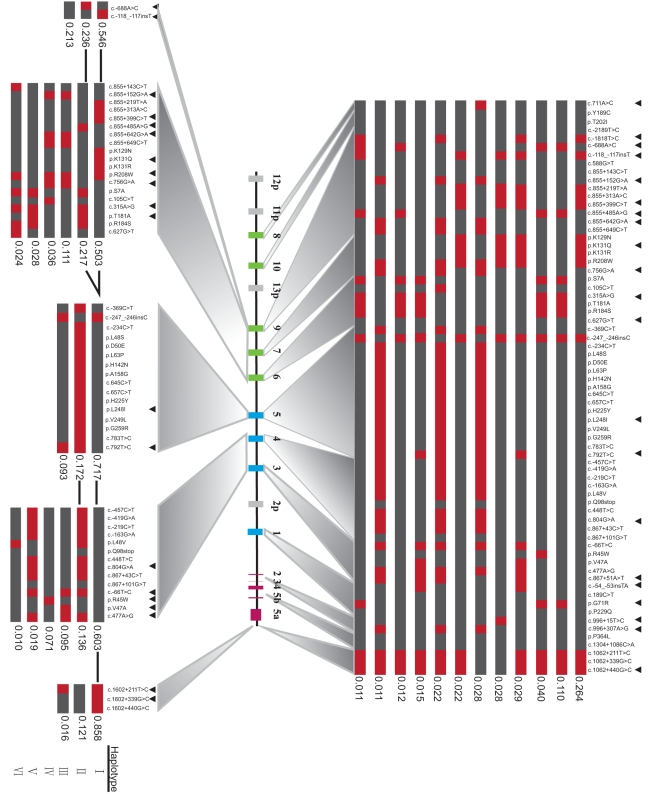
Haplotypes and tagSNPs in the *UGT1* locus and in each of the five haplotype block. The gray boxes in each haplotype represent the reference alleles of the AF297093.1 sequence, and the red ones represent the variants. Black triangle indicates tagSNPs. Thick lines between haplotypes of two blocks indicate a co-occurrence frequency >10%. Haplotypes with frequency of more than or equal to 1% are shown. TagSNPs are selected by using the STAMPA program, with a prediction accuracy >99% to represent all the polymorphisms in HWE in each haplotype.

We next identified haplotypes for the entire *UGT1* locus. We included rare polymorphisms with a frequency between 0.005 and 0.05 in addition to the polymorphisms with a frequency >0.05, because rare variants may play an important role in the etiology of complex diseases [41]. In this way, 337 haplotypes of the entire *UGT1* locus in this cohort were inferred, 12 of which exhibit a frequency >1%, representing 59.2% of all *UGT1* alleles (Fig. 2).

The most common haplotype of the *UGT1* locus has a frequency of 26.4% (Fig. 2). This haplotype contains two functional variants, 1A9*1b (c.−118_−117insT) and 1A7*1a (p. K129N/p.K131Q/p.K131R/p.R208W). The former has been associated with a modest increase in the promoter activity [34]; while the latter was associated with an approximately 6-fold higher glucuronosyltransferase activity *in vitro* [42]. Thus, this most common *UGT1* haplotype appears to encode 1A7 and 1A9 isozymes with the higher glucuronosyltransferase activity.

The second common haplotype has a frequency of 11% (Fig. 2). This haplotype includes one *1A9* promoter variant (c.−688A>C), one *1A9* intronic variant (c.855+485A>G), and three *1A6* coding variants 1A6*2 (p.S7A, p.T181A, p.R184S). The 1A6*2 allele has been associated with an increased glucuronidation activity [43]. This second haplotype also includes the 1A1*6 (p.G71R) allele, which exhibits the reduced enzymatic activity [44]. Thus, these *1A9*, *1A6*, and *1A1* alleles may have compensatory effects in this *UGT1* haplotype.

We used the STAMPA software to select tagSNPs in the *UGT1* locus and in five different blocks. We found that 21 SNPs in the *UGT1* locus can represent all of the 69 polymorphisms of frequency >0.5% with the accuracy of 99.03%. Thus, these 21 SNPs are tagSNPs of the *UGT1* locus (Fig. 2).

### Haplotypes Reconstruction and TagSNPs Selection for the Five LD Blocks

We also reconstructed haplotypes for the five LD blocks (Fig. 2). The haplotype diversity of each block is relatively limited in comparison with that of the entire locus. The haplotypes with the frequency >1% in each of the five blocks are shown in the Figure 2, representing 99.5%, 91.9%, 98.2%, 93.4%, and 99.5% of all chromosomes, respectively. The five LD blocks have 2, 9, 2, 5, and 2 tagSNPs with the prediction accuracy of 100%, 99.01%, 99.82%, 99.14%, and 99.41%, respectively (Fig. 2). The most common haplotypes of each block all have a frequency >50%. The differences in the haplotype diversity between the whole locus and the individual blocks suggest that there have been lots of recombination events between blocks.

### Haplotypes Reconstruction and TagSNPs Selection for Nine *UGT1* Variable Exons

**Figure 3:**
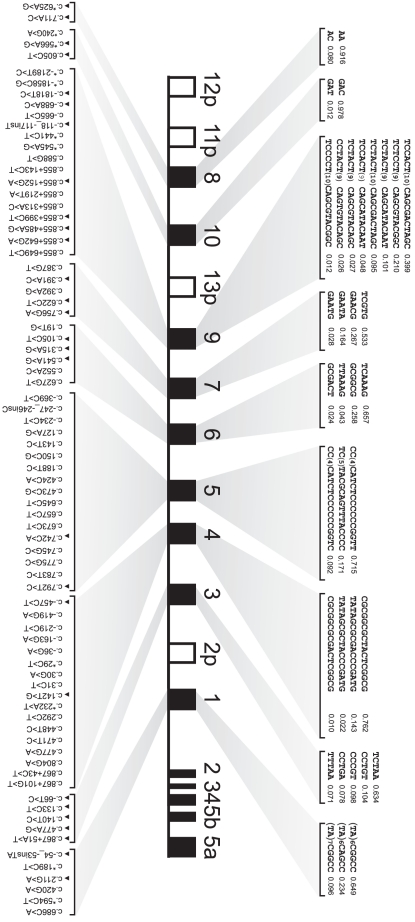
Haplotypes and tagSNPs in each of nine variable exons. All of the polymorphisms in HWE are included in the analysis. The haplotypes with frequency of more than or equal to 1% are shown. Black triangle indicates tagSNPs for each gene.

In the above analysis, the *UGT1A8*, *UGT1A10*, and *UGT1A1* genes were not found to belong to any blocks (Fig. 1B). Since it has been suggested that it is more reliable to identify tagSNPs for each *UGT1* gene than for haplotype blocks containing multiple genes [38], we also determined haplotypes and tagSNPs for each of the nine individual *UGT1* genes. We included 78 polymorphisms in HWE in this analysis, excluding the SNPs located within the constant exons and the 3′ UTR (Fig. 3). The haplotypes with a frequency >1% for each of the nine *UGT1* variable exons account for 99.6%, 99%, 91.9%, 99.2%, 98.2%, 97.8%, 93.7%, 98.5%, and 97.9% of all chromosomes, respectively (Fig. 3).

The *UGT1A9* gene has the most haplotype diversity, in which 48 haplotypes were identified and 8 with a frequency >1% (Fig. 3). The reference sequences of AF297093.1 [28] represent the most common haplotype of *UGT1A1*, *UGT1A3*, *UGT1A6*, *UGT1A8* and *UGT1A10* genes. The reference sequence allele of the *UGT1A9* variable exon only has a frequency of 2.7% (Fig. 3). Moreover, the reference sequence allele of the *UGT1A5* variable exon is not represented in the reconstructed *UGT1A5* haplotypes (data not shown).

The most common haplotype of *1A9* (39.9%) contains five variants of c.−1818T>C, c.855+219T>A, c.855+313A>C, c.855+399C>T, and c.−118_−117insT (1A9*1b). By contrast, the most common haplotype of *1A4* contains only one variant, c.471T>C (1A4*1b), with a frequency of 76.2%. The most common haplotype of *1A7*, 1A7*1a with a frequency of 53.3%, contains four variants: p.K129N, p.K131Q, p.K131R, and p.R208W.

Following the aforementioned procedure, we determined the tagSNPs in each of the nine *UGT1* variable exons. We found between 2 to 7 tagSNPs for each of the variable exons with a prediction accuracy >99% (Fig. 3).

### Comparison of Polymorphisms in Four Populations

**Figure 4:**
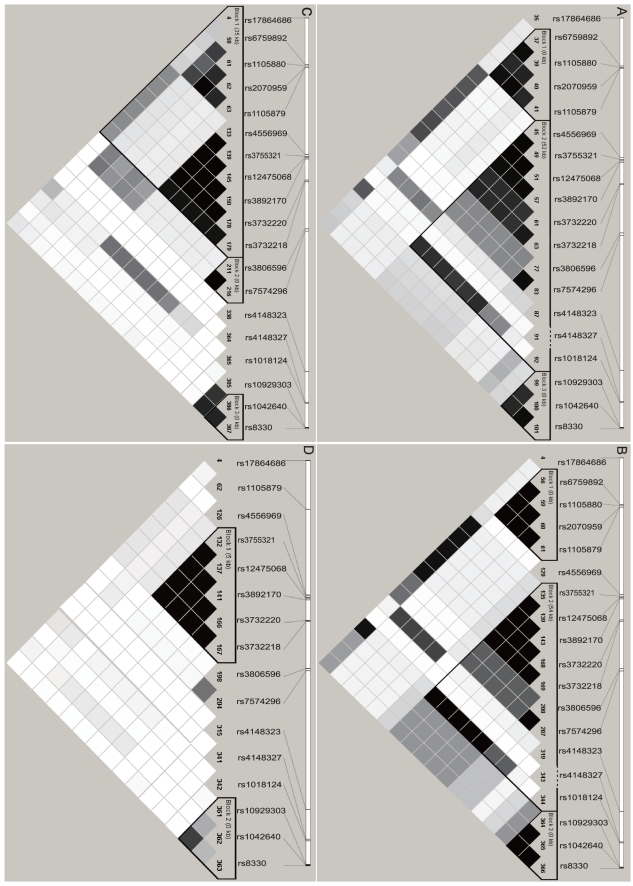
Comparsion of LD map of CHH with those of three HapMap populations. The extent of pairwise LD is represented by different shades of gray squares. The thick lines indicate the boundaries of haplotype blocks. The scale above each graph indicates the relative positions of the polymorphisms on chromosome 2. (**A**) Chinese (CHH, 253 unrelated individuals). (**B**) Japanese (JPT, 45 unrelated individuals). (**C**) Caucasian (CEU, 30 trios). (**D**) African (YRI, 30 trios).

To compare with the Chinese cohort, we downloaded genotyping data of the JPT, CEU, and YRI groups from the HapMap Database (http://hapmap.ncbi.nlm.nih.gov). We found that 19 polymorphisms are shared by CHH, JPT, and CEU, of which only 16 polymorphisms exist in the YRI population (Fig. 4).

The common SNPs of the *UGT1* locus in these four populations have ethnic-specific frequencies (Table 1). For example, *UGT1A3* −66T>C (rs3806596) has a frequency of 0.2–0.5 in CHH, JPT, and CEU, but is very rare (0.058) in YRI. Compared with the African and Caucasian populations, the SNP frequencies are more similar in the Asian populations (CHH and JPT). Many SNPs have similar frequencies in these two populations, e.g., p.L63P, p.A158G (1A5*2), and p.G259R of *1A5*; c.−419G>A, c.−163G>A, and c.471T>C (1A4*1b) of *1A4*; c.−66T>C and c.477A>G (1A3*1c) of *1A3*; and the intronic SNP c.996+307A>G (Table 1). Interestingly, the abundant SNP of *1A1* in Asian populations, p.G71R (1A1*6), has a much higher frequency in Chinese (0.241) (Table S2) than in Japanese (0.114) (Table 1).

**Table 1:** Comparison of SNP frequencies at the *UGT1* locus in four populations.

UGT1gene	rs Number (dbSNP database)	Variant ID	Populations			
			CHH	JPT	YRI	CEU
1A7	rs17864686	c.756G>A	0.170	0.205	0.051	0.242
1A6	rs6759892	p.S7A	0.300	0.148	0.433	0.375
	rs1105880	c.315A>G	0.290	0.148	0.441	0.310
	rs2070959	p.T181A	0.266	0.148	0.267	0.271
	rs1105879	p.R184S	0.286	0.148	0.325	0.308
	rs17863783	c.627G>T	0.024	0	0.167	0.033
1A5	rs5020121	c.−369C>T	0.189	n.c.	n.c.	0.125
	rs4556969	c.−234C>T	0.187	0.023	0.008	0.125
	rs3755321	p.L63P	0.191	0.193	0.125	0.125
	rs12475068	p.A158G	0.189	0.193	0.125	0.125
	rs3892170	p.G259R	0.183	0.193	0.125	0.125
1A4	rs3732221	c.−457C>T	0.190	n.c.	n.c.	0.114
	rs3732220	c.−419G>A	0.191	0.193	0.125	0.125
	rs3732218	c.−163G>A	0.194	0.193	0.125	0.117
	rs2011404	c.471T>C	0.998	1	1	0.847
1A3	rs3806596	c.−66T>C	0.291	0.273	0.058	0.417
	rs7574296	c.477A>G	0.289	0.273	0.100	0.417
1A1	rs4148323	p.G71R	0.241	0.114	0	0
Exon 2	rs4148327	c.996+15T>C	0.048	0.023	0	0
	rs1018124	c.996+307A>G	0.199	0.193	0.119	0.075
3′ UTR	rs10929303	c.1602+211T>C	0.875	0.841	0.533	0.792
	rs1042640	c1602+339G>C	0.860	0.841	0.797	0.784
	rs8330	c1602+440G>C	0.862	0.841	0.500	0.767

The common SNPs of the *UGT1* locus and their frequencies in the four populations are listed. The rs number and Variant ID are also shown.

CHH, Chinese in Henan province, China, 253 unrelated individuals.

n.c., not characterized.

### Comparison of LD Map in Four Populations

We performed an LD analysis by using 19 common polymorphisms of the CHH and JPT datasets. These two populations have highly similar LD maps (Fig. 4). For example, both populations have three similar LD Blocks (Fig. 4A,B). However, c.−234C>T (rs4556969) of *1A5* is within the second LD block in CHH but not in JPT (Fig. 4A,B). In addition, we also compared our data with the HapMap data of the Han Chinese. The results are overall very similar except that there exists a large linkage block in the Chinese HapMap data (data not shown).

For better comparison, we included the *1A1* polymorphisms 1A1*6 (rs4148323) and exon 2 c.996+15T>C (rs4148327) of *UGT1A1* in the LD analysis for the CEU and YRI populations despite the fact that their frequencies were zero in these two population (Table 1; Fig. 4C,D). Compared with CHH and JPT, the linkage pattern of highly-linked SNPs is similar in CEU (Fig. 4C). We excluded p.S7A (rs6759892), c.315A>G (rs1105880), and p.T181A (1A6*5) (rs2070959) of *1A6*, which are not in HWE, in the LD analysis for the YRI cohort. In comparison with CHH, JPT, and CEU, our results showed a very low level of the long-distance LD, consistent with more recombinations, in the *UGT1* locus in the YRI population (Fig. 4D).

### Haplotype Comparisons in Four Populations

**Figure 5:**
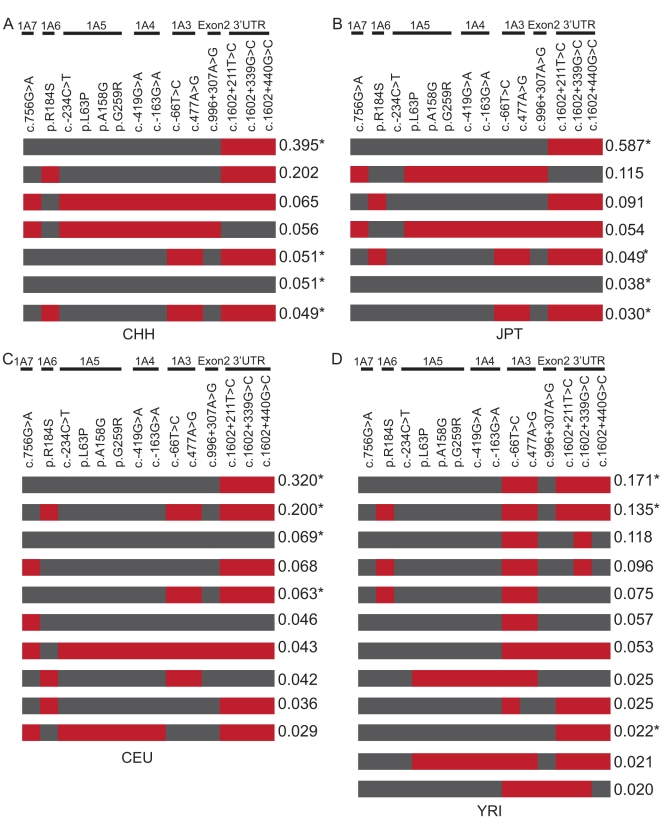
Haplotypes and their frequencies in CHH and three HapMap populations. Only the 14 polymorphisms shared by these four groups are included in the analysis. The haplotypes with frequency more than or equal to 2% are shown. The asterisk (*) indicates the common haplotypes shared by these four populations. The gray boxes in each haplotype represent the reference alleles of the AF297093.1 sequence, and the red ones represent the variants. (**A**) Chinese. (**B**) Japanese. (**C**) Caucasian. (**D**) African.

To compare haplotypes of the *UGT1* locus in the four populations, we reconstructed the *UGT1* haplotypes for each of the four population cohorts by using the 14 common polymorphisms in HWE with the Phase2.1.1 program. We listed the haplotypes with a frequency >2% (Fig. 5).

We observed that four common haplotypes of the *UGT1* locus are shared by these four populations. Each has a haplotype frequency >2% (Fig. 5, indicated by asterisks) with the exception of one with the frequency of 1.3% in YRI (data not shown). There are two additional common haplotypes in the CHH, JPT, and CEU populations (Fig. 5, and data not shown). In total, five haplotypes (>2%) with similar frequencies are shared between CHH and JPT, suggesting that these two populations are much closer. The most common haplotype in each of the four populations includes three polymorphisms of 3′ UTR (1A1*76, 1A1*78, and 1A1*79) (Fig. 5). We noticed that one functional polymorphism, i.e. p.R184S of *1A6* (1A6*9), resides in many high frequency haplotypes (Fig. 5).

We noted that there exists an ethnic specificity in the haplotype distribution of these four populations. For example, CHH has one specific haplotype with the frequency of 1.2% (data not shown). Moreover, JPT has one specific haplotype with the frequency of 5.4% (Fig. 5B). In addition, CEU has three specific haplotypes with the frequencies of 4.6%, 2.9% (Fig. 5C), and 1.5% (not shown). Finally, YRI has the most haplotype diversity (Fig. 5D). For example, YRI has eight specific haplotypes with the frequency >2%.

## Discussion

The UGT family proteins encoded by the *UGT1* gene cluster are the major drug-metabolizing enzymes, catalyzing about 35% of all phase II drug metabolizing reactions [3]. Single nucleotide polymorphisms of the *UGT1* gene cluster, which alter amino acids or change gene expression levels, have significant clinical phenotypes, such as variability in inter-individual drug efficacy and/or toxicity [26]. Previous studies have analyzed the genetic architecture of the *UGT1* gene cluster in the Caucasian, African, and Japanese populations [9], [37], [38], [45]. However, the SNPs and haplotypes of the entire *UGT1* gene cluster have not been analyzed in a large sample of the Chinese population.

Here, we resequenced all of the variable and constant exons and their surrounding regulatory noncoding regions of the entire *UGT1* gene cluster in 253 healthy Chinese individuals. We included the *UGT1A5* gene in our resequencing regions for completeness, even though this gene was not included in most other studies because of its low enzymatic activity and substrate uncertainty [9], [37], [38], [45]. We identified 15 novel polymorphisms in this Chinese cohort. We analyzed the polymorphism distribution, established the LD map, and reconstructed the haplotype patterns. This is the first report regarding the numerous genetic variations and their distribution attributes within the Chinese population.

We found 101 polymorphisms in the nine functional *UGT1* genes and their flanking sequences of about 17.7 kb of the *UGT1* cluster. The polymorphisms in this cluster are unusually abundant (5.7 SNPs per kb) because there is, on average, only one SNP per kb in the human genome [46]. In particular, there are currently 72 nonsynonymous SNPs in the nine coding variable regions (comprising about 7 kb) of the human *UGT1* cluster. This suggests that there may be an adaptive evolutionary force for selecting the molecular diversity in the *UGT1* cluster among individuals in humans. We previously found that the adaptive evolution plays an essential role for selecting diversified residues in the N-terminal domains of the nine functional human UGT1 enzymes [7]. By sampling a population of 253 Chinese individuals, our data confirm this initial observation and extend it to suggest that additional adaptive evolution for SNP diversity exists in the human *UGT1* locus of the phase II drug-metabolizing enzymes. Interestingly, there also exists the adaptive evolution of SNP diversity in the human *SULT1C2* locus, which also encodes a phase II drug-metabolizing enzyme [46], suggesting that the adaptive evolution may be a general phenomenon for enhancing the molecular diversity of the phase II drug-metabolizing enzymes. The gene clusters in the immune systems are known to have the adaptive evolution of SNP diversity and this diversity has inheritable influences on the expression regulation of the immune gene clusters [46]. It will be interesting to determine whether there are adaptive changes for polymorphisms in about 1 million-bp region of the three human neural *Pcdh* clusters.

We identified 11 novel rare polymorphisms in the *UGT1* gene cluster with the frequencies <0.5%. These rare polymorphisms may be important in the future analysis of *UGT1* inheritable diseases as well as the pharmacogenetic studies of drug metabolism. Increasingly, increasing evidence suggests that rare variants may be the causative factors of and contribute to multifactorial inheritance disease risks [41]. By contrast, common variants may only confer relatively small increments in disease risks [41]. Thus, we included rare SNPs (0.05> MAF >0.005) in the *UGT1* haplotype analysis, which potentially increases the power in future haplotype association studies.

The UGT1A1 protein is the only relevant bilirubin glucuronidating isozyme among members of the UGT1 protein family encoded by the human *UGT1* gene cluster [47]. To date, numerous polymorphisms of *UGT1A1* have been identified in association with human diseases of CNI, CNII, and GS [7], [15]. The polymorphic insertions in the promoter region of the human *UGT1A1* gene are associated with the efficiency of irinotecan metabolism [26]. The frequency of the allelic variant c.−54_−53insTA (1A1*28) (0.105) of the *UGT1A1* gene in the Chinese cohort (Table S2) is quite similar to that (0.143) in the healthy Taiwanese [48]. The coding polymorphism G71R of the human *UGT1A1* gene is associated with the efficacy of the jaundice phototherapy in infants [49], [50]. The frequency of this variant c.211G>A (1A1*6) (0.241, Table S2), which is associated with the serum bilirubin level in the Asian populations, is similar to previously reported [50]. Finally, we found the complete linkage of the *UGT1A4* intronic SNP c.867+101G>T (1A4*1d) with the *UGT1A1* c.686C>A (p.P229Q) (UGT1A1*27). Thus, we suggest that this *UGT1A4* intronic SNP can be used as a genotyping marker for the Gilbert Syndrome allele of UGT1A1*27.

We identified most of the polymorphisms previously reported in Asian populations [38]. The Asian population sample in this previous study included people from Southeast Asian countries, such as Philippines (4 individuals), Vietnam (4 individuals), and Thailand (3 individuals), in addition to China (17 individuals). This is consistent with the idea that Southeast Asian people may have migrated to East Asia in history [51]. However, we noted that some previously reported alleles, such as c.719C>T of *1A9*, c.211G>T and c.272G>C of *1A5*, c.173C>T, c.219A>C, and c.605C>T of *1A4* [38], were not observed in our study. In addition, we did not observe the two polymorphisms, p.P451L and Y486D of *UGT1A1*, previously reported in the Singaporean Chinese [49]. This suggests that there is a heterogeneous distribution of variants between Asian populations.

Our LD and haplotype analyses of the *UGT1* gene cluster demonstrate the ethnic specificity in the LD and haplotype patterns. For example, Block 9/7/6 and Block 3′ UTR are present in the Chinese, Japanese, and French-Canadian populations, while Block 5 and Block 4/3 are only observed in the Chinese population. The same haplotypes coexist in different samples with different frequencies; and different populations have their own specific haplotypes. Five haplotypes are shared by the CHH (Chinese), JPT (Japanese), CEU (Caucasian), and YRI (African) groups. However, each of these four populations has its own set of haplotypes (Fig. 5).

The LD map, haplotype block determination, and haplotype reconstruction are greatly dependent on the parameters chosen for the analyses, such as the threshold of SNP frequency and the algorithm used. The cutoff value of SNP frequency influences the LD pattern of the *UGT1* locus. For example, we did not observe the close linkage of the 686G>A (1A1*27) with (TA)6/7 in the *UGT1A1* gene, which was previously reported in Taiwanese [48]. By contrast, we observed that *UGT1A5* and *UGT1A4* are in separate blocks in our Chinese cohort (Fig. 1); However, it is linked in one block in a small sample of 50 Korean individuals [52]. Finally, when variants *1A9* c.855+143C>T and *1A6* c.627G>T, both having frequencies below 0.050 (Table S2), were included in the LD analysis, the Block 9/7/6 boundary was altered (not shown).

Compared with individual SNP markers, haplotype, which is the linked combination of polymorphisms, has the greater power to provide more useful information on genotype-phenotype analyses [27]. However, although haplotypes carry more information than SNPs, there are limitations of computational approaches for reconstructing haplotypes and for determining their frequencies. In this study, we used the *PHASE2.1.1* computer program, which has previously been shown to have a low error rate in the prediction of haplotypes [32], to reconstruct haplotypes in the Chinese population. Moreover, the relative large sample size of 253 individuals being examined in this study also decreases the error rate in the reconstruction.

We expect that the sets of tagSNPs for each LD block and for each gene identified here could be used for selective SNP genotyping and for inferring all of the non-typed SNPs at a considerable savings in cost [53]. Therefore, the tagSNPs identified here in the *UGT1* gene cluster are anticipated to provide a solid foundation for future pharmacogenomic studies. In summary, the genetic variation and haplotype architecture gained from this study should lay a fundamental basis for the prognosis of metabolism diseases as well as for future genomic applications, including the individualized medicine.

## Supporting Information

Table S1 **List of the primers for PCR amplification and sequencing.** List of all of the primers used to amplify and to sequence each of the 12 UGT1 regions is shown. The size of PCR products and the annealing temperature for each PCR reaction are also shown. The usage of the primers for PCR (P) and for sequencing (S) is indicated. In three cases, a second primer was used for sequencing. F, forward primer; R, reverse primer.
(DOC)

Table S2 **SNPs and their frequencies identified in Chinese population.** The 101 polymorphisms identified in the Chinese population are listed. The dbSNP Submitter SNP (ss) accession numbers for 15 novel SNPs are also shown. The positions for all of 101 SNPs are according to the finished February 2009 human reference sequence assembly (GRCh37). The corresponding allele frequencies are shown in the right column.
(DOC)
