# Supplementary material for: Genetic Variations and Haplotype Diversity of the UGT1 Gene Cluster in the Chinese Population
Source: PLoS One. 2012 Apr 13;7(4):e33988. doi: 10.1371/journal.pone.0033988 (PMC3325998; doi:10.1371/journal.pone.0033988)
Supplement: Table S1 — List of the primers for PCR amplification and sequencing. List of all of the primers used to amplify and to sequence each of the 12 UGT1 regions is shown. The size of PCR products and the annealing temperature for each PCR reaction are also shown. The usage of the primers for PCR (P) and for sequencing (S) is indicated. In three cases, a second primer was used for sequencing. F, forward primer; R, reverse primer. (DOC) [file pone.0033988.s001.doc]

**Supplemental Table 1. List of primers** for PCR amplification and sequencing

| Gene | PCR(p)/Seq(S) Primers | Sequences | PCR product  size (bp) | Annealing Temperature (℃) |
| --- | --- | --- | --- | --- |
| UGT1A8 | F/P/S | TAAACTCACGTTACAGCACTGG | 1334 | 61 |
|  | R/P/S | CACACCTTCAAGAAGGGCAGTT |  |  |
| UGT1A10 | F/P/S | ACTCACTTGCAGCGTGCTCTC | 1277 | 65 |
|  | R/P/S | TGATGAGTACATGAATTCGCAC |  |  |
|  | F2/S | AAATACACGCCCTCTATTGG |  |  |
| UGTA19 | F/P/S | GTAGGTCTTTTACATTTCC | 943 | 50 |
|  | R/P/S | AATGGAGGACAATCAAGAG |  |  |
|  | F/P/S | AGAATGTGCAAGTTGAGCGGTC | 805 | 62 |
|  | R/P/S | CAGACACACACATAGAGGAAGG |  |  |
|  | F/P/S | GTATTTCTCCCACCTACTGTAT | 966 | 57 |
|  | R/P/S | AGGTGAAGTATTCTTAAGGTGC |  |  |
|  | F/P/S | AGCACCTTAAGAATACTTCACC | 745 | 58 |
|  | R/P/S | TGCCAATCCTTCTCAATTCATG |  |  |
| UGT1A7 | F/P/S | GAGGGCAGGTTCTATCTG | 963 | 61 |
|  | R/P/S | GCTAAAGGGGAGATAACTTACC |  |  |
| UGT1A6 | F/P/S | GTCAGACAGAAGTTGTGGGTAACCTG | 1621 | 59 |
|  | R/P/S | CACTTCAGCCTCAGGTCTCCTATGTG |  |  |
| UGT1A5 | F/P/S | GGATGTGCTGTGTTACCCATA | 1527 | 58 |
|  | R/P/S | ACCCTGAACTGCACTACCATTG |  |  |
| UGT1A4 | F/P/S | AGATAGCCAGCCTGAACACTC | 1592 | 60 |
|  | R/P/S | CTTCTTCCTCTCAGTGACCAC |  |  |
| UGT1A3 | F/P/S | AGTGAGCACAGGGTCAGACGT | 1466 | 62 |
|  | R/P/S | TCCAGGATGGATCAGTTCCA |  |  |
|  | F2/S | ACGTGTTTTTCAAGATAGTC |  |  |
| UGT1A1 | F/P/S | CAGTCAAACATTAACTTGGTGT | 1051 | 58 |
|  | R/P/S | TGCTTGCTCAGCATATATCT |  |  |
|  | R2/S | ACAACGAGGCGTCAGGTGCT |  |  |
| Exons 2,3,4 | F/P/S | CTCTATCTCAAACACGCATG | 1628 | 54 |
|  | R/P/S | CAACGCTATTAAATGCTACGTA |  |  |
| Exon 5b | F/P/S | AAGAAGTGGTGGCCAGTGGTAG | 564 | 64 |
|  | R/P/S | CTGGGCACATGATGCTCAGGTT |  |  |
| Exon 5a | F/P/S | ATCTGGTAGTCTTCTTAAGCAG | 1273 | 57 |
|  | R/P/S | TTATGCATGCACACGCAATGAA |  |  |

List of all of the primers used to amplify and to sequence each of the 12 *UGT1* regions is shown. The size of PCR products and the annealing temperature for each PCR reaction are also shown. The usage of the primers for PCR (P) and for sequencing (S) is indicated. In three cases, a second primer was used for sequencing. F, forward primer; R, reverse primer.
