# Supplementary material for: Genetic Variations and Haplotype Diversity of the UGT1 Gene Cluster in the Chinese Population
Source: PLoS One. 2012 Apr 13;7(4):e33988. doi: 10.1371/journal.pone.0033988 (PMC3325998; doi:10.1371/journal.pone.0033988)
Supplement: Table S2 — SNPs and their frequencies identified in Chinese population. The 101 polymorphisms identified in the Chinese population are listed. The dbSNP Submitter SNP (ss) accession numbers for 15 novel SNPs are also shown. The positions for all of 101 SNPs are according to the finished February 2009 human reference sequence assembly (GRCh37). The corresponding allele frequencies are shown in the right column. (DOC) [file pone.0033988.s002.doc]

**Supplemental Table 2. SNPs and their frequencies identified in Chinese population**

| UGT1 gene | Rs or Ss Number | Allele | Genomic | Coding DNA | Protein | MAF^g^ |
| --- | --- | --- | --- | --- | --- | --- |
|  | (dbSNP database) | name^b^ | Location^c^ | nucleotide numbering^d^ | change^f^ |  |
| 1A8 | rs1042597 | 1A8*2 | 234526871 | c.518C>G | p.A173G | 0.443 |
|  | ss469324745 |  | 234526978 | c.*625A>G^e^ | p.N209D | 0.004 |
|  | rs1126805 |  | 234527064 | c.711A>C |  | 0.083 |
|  | rs1042605 | 1A8*1b | 234527118 | c.765A>G |  | 0.049 |
| 1A10 | ss469324746 |  | 234545408 | c.*240G>A |  | 0.004 |
|  | ss469324747 |  | 234545590 | c.*422C>G | p.S141C | 0.004 |
|  | ss469324748 |  | 234545734 | c.*566A>G | p.Y189C | 0.006 |
|  | rs58704432 | 1A10*6 | 234545773 | c. 605C>T | p. T202I | 0.012 |
|  | rs17854828 |  | 234545861 | c.693C>T |  | 0.062 |
| 1A9 | ss469324749 |  | 234578392 | c.*-2189T>C |  | 0.026 |
|  | rs6731242 |  | 234578693 | c.-1887T>G |  | 0.045 |
|  | ss469324750 |  | 234578722 | c.*-1859C>G |  | 0.002 |
|  | rs13418420 |  | 234578762 | c.-1818T>C |  | 0.502 |
|  | rs3806598 |  | 234579892 | c.-688A>C |  | 0.241 |
|  | rs10176426 |  | 234579915 | c.-665C>T |  | 0.004 |
|  | rs2741045 |  | 234580140 | c.-441T>C |  | 0.970 |
|  | rs2741046 |  | 234580249 | c.-332C>T |  | 0.970 |
|  | rs35426722 | 1A9*1b | 234580454 | c.-118_-117insT |  | 0.551 |
|  | ss469324751 |  | 234581021 | c.*441C>T |  | 0.004 |
|  | ss469324752 |  | 234581125 | c.*545A>G | p.Q182R | 0.002 |
|  | --------- ^a^ |  | 234581168 | c.588G>T |  | 0.006 |
|  | rs45554333 |  | 234581578 | c.855+143C>T |  | 0.026 |
|  | rs4663871 |  | 234581587 | c.855+152G>A |  | 0.166 |
|  | rs2741047 |  | 234581654 | c.855+219T>A |  | 0.530 |
|  | rs2741048 |  | 234581748 | c.855+313A>C |  | 0.530 |
|  | rs2741049 |  | 234581834 | c.855+399C>T |  | 0.530 |
|  | rs7349250 |  | 234581920 | c.855+485A>G |  | 0.243 |
|  | rs2602376 |  | 234582051 | c.855+616T>C |  | 0.970 |
|  | rs17862856 |  | 234582077 | c.855+642G>A |  | 0.166 |
|  | rs17862857 |  | 234582084 | c.855+649C>T |  | 0.174 |
| 1A7 | rs17868323 |  | 234590970 | c.387G>T | p.K129N | 0.536 |
|  | rs17863778 |  | 234590974 | c.391A>C | p.K131Q | 0.536 |
|  | rs17868324 | 1A7*11 | 234590975 | c.392A>G | p.K131R | 0.538 |
|  | rs11692021 | 1A7*4 | 234591205 | c.622C>T | p.R208W | 0.727 |
|  | rs17864686 |  | 234591339 | c.756G>A |  | 0.170 |
| 1A6 | rs12476197 | 1A6*1c | 234601224 | c.-427G>C |  | 0.259 |
|  | rs6759892 |  | 234601669 | c.19T>G | p.S7A | 0.300 |
|  | rs45535938 | 1A6*1e | 234601755 | c.105C>T |  | 0.044 |
|  | rs1105880 |  | 234601965 | c.315A>G |  | 0.290 |
|  | rs2070959 | 1A6*5 | 234602191 | c.541A>G | p.T181A | 0.266 |
|  | rs1105879 | 1A6*9 | 234602202 | c.552A>C | p.R184S | 0.286 |
|  | rs17863783 |  | 234602277 | c.627G>T |  | 0.024 |
| 1A5 | rs5020121 |  | 234621269 | c.-369C>T |  | 0.189 |
|  | rs5839490 |  | 234621391 | c.-247_-246insC |  | 0.189 |
|  | rs4556969 |  | 234621404 | c.-234C>T |  | 0.187 |
|  | rs45441297 |  | 234621764 | c. 127A>G | p.S43G | 0.002 |
|  | rs3755323 |  | 234621780 | c.143T>C | p.L48S | 0.187 |
|  | rs3755322 |  | 234621787 | c.150C>G | p.D50E | 0.187 |
|  | rs3755321 |  | 234621825 | c.188T>C | p.L63P | 0.191 |
|  | rs3755320 |  | 234622061 | c.424C>A | p.H142N | 0.189 |
|  | rs12475068 | 1A5*2 | 234622110 | c.473C>G | p.A158G | 0.189 |
|  | rs17868333 |  | 234622282 | c.645C>T |  | 0.189 |
|  | rs17863790 |  | 234622294 | c.657C>T |  | 0.189 |
|  | rs17862867 | 1A5*3 | 234622310 | c.673C>T | p.H225Y | 0.189 |
|  | rs2012736 |  | 234622379 | c.742C>A | p.L248I | 0.189 |
|  | rs17862868 |  | 234622382 | c.745G>C | p.V249L | 0.183 |
|  | rs3892170 |  | 234622412 | c.775G>C | p.G259R | 0.183 |
|  | rs17862869 |  | 234622420 | c.783T>C |  | 0.183 |
|  | rs2012734 |  | 234622429 | c.792T>C |  | 0.272 |
| 1A4 | rs3732221 |  | 234627010 | c.-457C>T |  | 0.190 |
|  | rs3732220 |  | 234627048 | c.-419G>A |  | 0.191 |
|  | rs3732219 |  | 234627248 | c.-219C>T |  | 0.188 |
|  | rs3732218 |  | 234627304 | c.-163G>A |  | 0.194 |
|  | --------- | 1A4*1h | 234627431 | c.-36G>A |  | 0.002 |
|  | ss469324753 |  | 234627495 | c.*29C>T | p.P10L | 0.002 |
|  | --------- | 1A4*1e | 234627496 | c.30G>A |  | 0.002 |
|  | rs3892221 | 1A4*4 | 234627497 | c.31C>T | p.R11W | 0.004 |
|  | rs2011425 | 1A4*3b | 234627608 | c.142T>G | p.L48V | 0.178 |
|  | ss469324754 |  | 234627698 | c.*232A>T | p.T78S | 0.004 |
|  | --------- |  | 234627758 | c.292C>T | p.Q98stop | 0.008 |
|  | rs12468274 |  | 234627914 | c.448T>C |  | 0.189 |
|  | rs2011404 | 1A4*1b | 234627937 | c.471T>C |  | 0.998 |
|  | rs45467894 |  | 234627943 | c.477G>A |  | 0.004 |
|  | rs3732217 |  | 234628270 | c.804G>A |  | 0.190 |
|  | rs2011219 |  | 234628376 | c.867+43C>T |  | 0.188 |
|  | --------- | 1A4*1d | 234628434 | c.867+101G>T |  | 0.008 |
| 1A3 | rs3806596 |  | 234637707 | c.-66T>C |  | 0.291 |
|  | rs28898617 |  | 234637789 | c.17A>G | p.Q6R | 0.107 |
|  | rs3821242 |  | 234637803 | c.31T>C | p.W11R | 0.332 |
|  | rs6706232 | 1A3*1d | 234637853 | c.81G>A |  | 0.370 |
|  | rs45625338 |  | 234637905 | c.133C>T | p.R45W | 0.071 |
|  | rs6431625 | 1A3*10a | 234637912 | c.140T>C | p.V47A | 0.103 |
|  | rs7574296 | 1A3*1c | 234638249 | c.477A>G |  | 0.289 |
|  | rs2361501 |  | 234638690 | c.867+51A>T |  | 0.208 |
| 1A1 | rs34815109 | 1A1*28 | 234668881 | c.-54_-53insTA |  | 0.105 |
|  | ss469324755 |  | 234669122 | c.*189C>T |  | 0.008 |
|  | rs4148323 | 1A1*6 | 234669144 | c.211G>A | p.G71R | 0.241 |
|  | --------- |  | 234669353 | c.420G>A |  | 0.002 |
|  | ss469324756 |  | 234669527 | c.*594C>T |  | 0.002 |
|  | rs35350960 | 1A1*27 | 234669619 | c.686C>A | p.P229Q | 0.008 |
| Exon 2 | rs4148327 |  | 234675826 | c.996+15T>C |  | 0.048 |
|  | rs1018124 |  | 234676118 | c.996+307A>G |  | 0.199 |
|  | rs34082659 |  | 234676409 | c.996+598C>T |  | 0.004 |
|  | rs2302538 |  | 234676413 | c.996+602T>C |  | 0.044 |
| Exon 4 | rs34946978 |  | 234676872 | c.1091C>T | p.P364L | 0.010 |
|  | ss469324757 |  | 234678096 | c.*1304+1011T>C |  | 0.004 |
|  | ss469324758 |  | 234678171 | c.*1304+1086C>A |  | 0.014 |
| 3’ UTR | ss469324759 |  | 234681393 | c.*1602+188T>G |  | 0.002 |
|  | rs10929303 | 1A1*76 | 234681416 | c.1602+211T>C |  | 0.875 |
|  | rs1042640 | 1A1*78 | 234681544 | c.1602+339G>C |  | 0.860 |
|  | rs8330 | 1A1*79 | 234681645 | c.1602+440G>C |  | 0.862 |

The 101 polymorphisms identified in the Chinese population are listed. The dbSNP Submitter SNP (ss) accession numbers for 15 novel SNPs are also shown. The positions for all of 101 SNPs are according to the finished February 2009 human reference sequence assembly (GRCh37). The corresponding allele frequencies are shown in the right column.

^a^Rs number not registered in the dbSNP database.

^b^According to the UGT Allele Nomenclature website (www.ugtallels.ulaval.ca).

^c^With reference to Ensembl Homo Sapiens version 64.37 (GRCh37) Chromosome 2.

^d^cDNA numbering with +1 corresponding to the first nucleotide of the ATG translation initiation codon in the reference mRNA sequence. For the 3’ UTR polymorphisms, numbering starts at the first nucleotide after the stop codon of exon 5a.

^e^novel polymorphisms.

^f^Amino acid numbering with +1 corresponding to the first residue Methionine (Met) of the polypeptide chain.

^g^Minor allele frequency.
